# Evaluation of the Impact of Different Pain Medication and Proton Pump Inhibitors on the Osteogenic Differentiation Potential of hMSCs Using ^99m^Tc-HDP Labelling

**DOI:** 10.3390/life11040339

**Published:** 2021-04-11

**Authors:** Tobias Grossner, Uwe Haberkorn, Tobias Gotterbarm

**Affiliations:** 1 Trauma Surgery and Paraplegiology, Clinic for Orthopedics and Trauma Surgery, Center for Orthopedics, University Hospital Heidelberg, 69120 Heidelberg, Germany; 2 Department of Nuclear Medicine, University Hospital Heidelberg, 69120 Heidelberg, Germany; uwe.haberkorn@med.uni-heidelberg.de; 3 Clinical Cooperation Unit Nuclear Medicine, Deutsches Krebsforschungszentrum (DKFZ), 69120 Heidelberg, Germany; 4 Translational Lung Research Center Heidelberg (TLRC), German Center for Lung Research (DZL), 69120 Heidelberg, Germany; 5 Department of Orthopedics and Traumatology, Kepler University Hospital, 4020 Linz, Austria; tobias.gotterbarm@kepleruniklinikum.at

**Keywords:** mesenchymal stem cells, osteogenic differentiation, proton pump inhibitors, NSASIDs, ^99m^Tc-HDP labeling, hydroxyapatite, metamizole

## Abstract

First-line analgetic medication used in the field of musculoskeletal degenerative diseases, like Nonsteroidal anti-inflammatory drugs (NSAIDs), reduces pain and prostaglandin synthesis, whereby peptic ulcers are a severe adverse effect. Therefore, proton pump inhibitors (PPI) are frequently used as a concomitant medication to reduce this risk. However, the impact of NSAIDs or metamizole, in combination with PPIs, on bone metabolism is still unclear. Therefore, human mesenchymal stem cells (hMSCs) were cultured in monolayer cultures in 10 different groups for 21 days. New bone formation was induced as follows: Group 1 negative control group, group 2 osteogenic differentiation media (OSM), group 3 OSM with pantoprazole (PAN), group 4 OSM with ibuprofen (IBU), group 5 OSM with diclofenac (DIC), group 6 OSM with metamizole (MET), group 7 OSM with ibuprofen and pantoprazole (IBU + PAN), group 8 OSM with diclofenac and pantoprazole (DIC + PAN), group 9 OSM with metamizole and pantoprazole (MET + PAN) and group 10 OSM with diclofenac, metamizole and pantoprazole (DIC + MET + PAN). Hydroxyapatite content was evaluated using high-sensitive radioactive ^99m^Tc-HDP labeling. Within this study, no evidence was found that the common analgetic medication, using NSAIDs alone or in combination with pantoprazole and/or metamizole, has any negative impact on the osteogenic differentiation of mesenchymal stem cells in vitro. To the contrary, the statistical results indicate that pantoprazole alone (group 3 (PAN) (*p* = 0.016)) or diclofenac alone (group 5 (DIC) (*p* = 0.008)) enhances the deposition of minerals by hMSCS in vitro. There is an ongoing discussion between clinicians in the field of orthopaedics and traumatology as to whether post-surgical (pain) medication has a negative impact on bone healing. This is the first hMSC in vitro study that investigates the effects of pain medication in combination with PPIs on bone metabolism. Our in vitro data indicates that the assumed negative impact on bone metabolism is subsidiary. These findings substantiate the thesis that, in clinical medicine, the patient can receive every pain medication needed, whether or not in combination with PPIs, without any negative effects for the osteo-regenerative potential.

## 1. Introduction

In an aging population, the impact of musculoskeletal degenerative diseases (e.g., arthrosis and osteoporosis) and trauma is an enormous burden for healthcare systems [1]. At a certain point of treatment, nearly all patients who consult a doctor or physiotherapist due to a musculosceletal disorder are prescribed pain medication [2,3].

Usually, the pain medication strategy follows recommendations in guidelines published by large national societies like the “American Pain Society” [4] or the “Agreement of the Professional Association of German Anesthesiologists and the Professional Association of German Surgeons for the Organization of Postoperative Pain Therapy” [5] or the -S3- guideline for long-term application for opioids (LONTS) [6]. Interestingly, the WHO “Web statement on pain management guidance” states that the WHO is currently reviewing and updating its guidelines and policy documents regarding pain management.

Non-opioid drugs are the backbone of any adequate pain medication concept. Most of these drugs are NSAIDs (nonsteroidal anti-inflammatory drugs). Examples include acetylsalicylic acid (e.g., Aspirin^®^) (Bayer AG, Monheim am Rhein, Germany), ibuprofen (e.g. Nurofen^®^) (Reckitt Benckiser Deutschland GmbH, Heidelberg, Germany) and diclofenac (e.g., Voltaren^®^) (GlaxoSmithKline Consumer Healthcare GmbH & Co. KG, Munich, Germany) [2]. The anti-inflammatory potential of these drugs reduces symptoms caused by the inflammatory component in pathologies like activated arthrosis and post-surgical inflammatory response [7]. An additional feature of NSAIDs (e.g., diclofenac and ibuprofen) is their ability to reduce heterotopic ossifications after total hip arthroplasty and acetabular fracture treatment [8,9,10,11,12]. Non-selective NSAIDs inhibit COX-1 and COX-2, while selective NSAIDs only inhibit COX-2. Both pathways suppress the synthesis of prostaglandins, an important mediator for inflammation. This suppression upregulates the production of gastric acid [13] which causes one of the most severe side effects of these drugs, namely gastrointestinal ulcers. Proton pump inhibitors (PPIs) are therefore frequently used as an accompanying medication during treatment with NSAIDs to prevent such gastrointestinal side effects [13]. However, non-NSAIDs are also widely used for first-line pain medication. Here, metamizole (e.g., Novalgin^®^) (Sanofi-Aventis Deutschland GmbH, Frankfurt am Main, Germany) and paracetamol (e.g., Tylenol^®^) (Johnson & Johnson Consumer Inc, Fort Washington, PA 19034, USA) are the most common agents. While their analgetic potential is at least as high as NSAIDs, their anti-inflammatory potential is comparably less [3,7].

The potential relationship between PPIs and fracture risk is disputed and the recent data analysis from the Trøndelag Health Study (HUNT) study did not reveal any fracture risk associated with the use of PPIs [14,15]. While little in vitro data is available on the impact of PPIs on osteoblast and osteoclast-like cells, some pilot projects assume a negative impact of PPIs on bone metabolism [16], while other studies show no negative effects on primary human osteoblasts [17]. There are indications that bone degradation and resorption are inhibited by PPIs due to reduced osteoclast activity [18]. Data about the impact of NSAIDs on bone metabolism and bone healing are inconclusive. Recent systematic reviews found no consensus regarding the risks and benefits of treating musculoskeletal disorder patients with NSAIDs and the impact of these drugs on bone metabolism [19], while another review assumes the absence of robust clinical and scientific data [20]. There is also a lack of data concerning the effects of non-NSAIDs like metamizole on bone metabolism and no data available concerning the effects of metamizole in combination with NSAIDs and PPIs. Furthermore, no experimental data is available for evaluating the effects of standard pain medication (NSAIDS and non-NSAIDs) in combination with PPI therapy on the osteogenic potential of human mesenchymal stem cells in vitro.

Recently, ^99m^Tc-HDP labeling has been employed as a highly sensitive, non-destructive and effective method to quantify the amount of hydroxyapatite within osteogenic-induced monolayer cell cultures of human mesenchymal stem cells This method has been positively verified by correlation analysis with the standard marker for osteogenesis in vitro in previous studies (quantitative Alizarin red staining, von Kossa staining, scanning electron microscope with energy-dispersive X-ray spectroscopy, inductively coupled plasma mass spectrometry for absolute calcium) [21]. Here, the radioactive tracer ^99m^Tc bound to a polyphosphonate binds to inorganic mineral in areas with high osteogenic activity, e.g., hydroxyapatite deposition, by chemisorption. As ^99m^Technetium decays by gamma decay, emitting energy with 140 keV, these counts can be easily detected and registered with a gamma camera. The amount of bound tracer is proportional to the amount of hydroxyapatite deposition and to the osteogenic potential of the experimental cell culture setting [21].

This experimental in vitro study, therefore, seeks to discover the effect of clinically used pain medication on new bone formation by human mesenchymal stem cells (hMSCs) to evaluate if single and combined non-opioid analgetic drugs exert any impact on the osteogenic response of human mesenchymal stem cells and if the accompanying PPI medication exert any influence on this impact.

## 2. Results

### 2.1. ^99m^Tc-HDP Labelling

The human donors exhibited marginal variability regarding individual osteogenic potential for the uptake of the tracer within each group. The negative control group (group 1, NCG) showed almost no uptake (mean 327 counts/180 s) while the highest mean uptake was seen within group 5 treated with diclofenac (mean 61,047 counts/180 s). The second highest uptake was discovered for the group treated with pantoprazole (group 3, mean 60,333 counts/180 s), followed by group 4 treated with ibuprofen (mean 59,124 counts/180 s). The mean uptake within the other groups was between 55,939 and 58,624 counts/180 s.

The tracer uptake was at least 171-fold higher within the osteogenic groups (groups 2–10) compared to the negative control group (group 1) with the lowest uptake (group 10, DIC + MET + PAN) 55,939 counts/180 s), while it was 186-fold higher for the osteogenic group with the highest uptake (group 5 DIC, 61,047 counts/180 s).

A complete overview regarding the tracer uptake is shown in Table 1.

### 2.2. Statistical Results

The Kolmogorov-Smirnov test for normal distribution was performed prior to further data analysis and revealed a normal distribution for tracer uptake within each of the ten groups (*p* ≤ 0.001).

The first Analysis of variance (ANOVA) analysis revealed a highly significant effect of the osteogenic media for all osteogenic groups (2, 3, 4 and 7) versus the negative control group (group 1) with *p* ≤ 0.001, revealing a successful osteogenic differentiation. Additionally, a significantly higher uptake was noted for the group where only pantoprazole (group 3) was added versus the group in which only the standard osteogenic media (group 2) was used (*p* ≤ 0.05). No significantly higher uptake was noted for the group in which IBU (group 4) and IBU + PAN (group 7) was added (*p* = 0.135 and *p* = 0.284) versus the standard osteogenic group (group 2). Therefore, no significant negative effect regarding the osteogenesis could be shown by adding ibuprofen or ibuprofen and pantoprazole to the cell culture media, while adding just pantoprazole alone resulted in a significant enhancement of the tracer uptake.

The second ANOVA analysis also revealed a highly significant effect of the osteogenic media for all osteogenic groups (2, 3, 5 and 8) versus the negative control group (group 1) with *p* ≤ 0.001, and so also revealed successful osteogenic differentiation. Additionally, a significantly higher uptake was noted for the group where only pantoprazole (group 3) was added versus the group in which only the standard osteogenic media (group 2) was used (*p* ≤ 0.05). Additionally, there was a significantly higher uptake (*p* ≤ 0.01) of the tracer in group 5. Here, only diclofenac was added to the standard osteogenic media (group 2). No significantly higher uptake was noted for the group in which DIC + PAN (group 8) was added (*p* = 1.00) versus the standard osteogenic group (group 2). Therefore, a significant effect regarding osteogenesis could be shown by adding pantoprazole or diclofenac to the cell culture media, while adding diclofenac and pantoprazole did not result in a significant enhancement of the tracer uptake.

The third ANOVA analysis also revealed a highly significant effect of the osteogenic media for all osteogenic groups (2, 3, 6 and 9) versus the negative control group (group 1) with *p* ≤ 0.001, which also revealed a successful osteogenic differentiation under these conditions. Additionally, a significantly higher uptake was noted for the group where only pantoprazole (group 3) was added versus the group in which only the standard osteogenic media (group 2) was used (*p* ≤ 0.05). No significantly higher uptake was noted for the groups in which MET (group 6) and MET + PAN (group 9) were added (*p* = 0.925 and *p* = 0.998) versus the standard osteogenic group (group 2). Therefore, no significant effect regarding osteogenesis could be shown by adding either metamizole or metamizole and pantoprazole to the culture media, whereas adding just pantoprazole led to a significant enhancement of the tracer uptake.

Likewise, the fourth ANOVA analysis revealed a highly significant effect of the osteogenic media for all osteogenic groups (2, 3 and 10) versus the negative control group (group 1) with *p* ≤ 0.001. Additionally, in this analysis, a significantly higher uptake was noted for the group where only pantoprazole (group 3) was added versus the group in which only the standard osteogenic media (group 2) was used (*p* = 0.016), while no significant effect was shown by adding a combination of DIC + MET + PAN (group 10) to the cell culture media compared to the standard osteogenic media (group 2) (*p* = 1).

In summary, no significant effect regarding osteogenesis could be shown by adding metamizole in combination with diclofenac and pantoprazole to the cell culture media, while adding just pantoprazole led to a significant enhancement of the tracer uptake.

**Figure 1:**
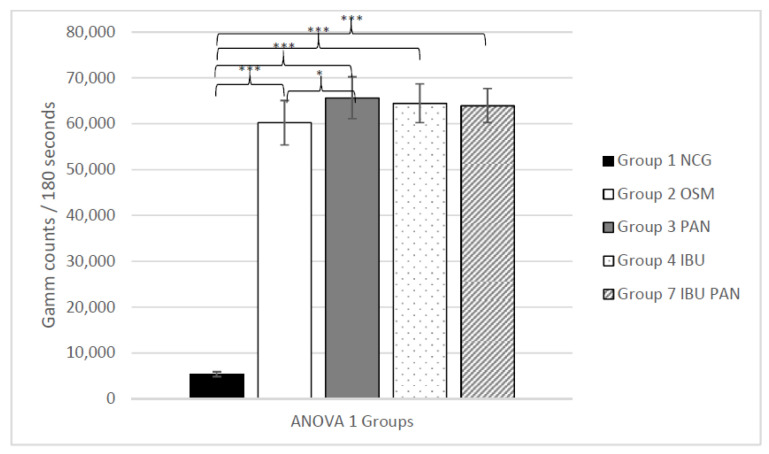
ANOVA 1, analyzing the ^99m^TC-HDP uptake (counts)/180 s for groups 1–4 and 7 +/− standard deviation. Statistical significance is indicated by *** = *p* ≤ 0.001; * = *p* ≤ 0.05.

**Figure 2:**
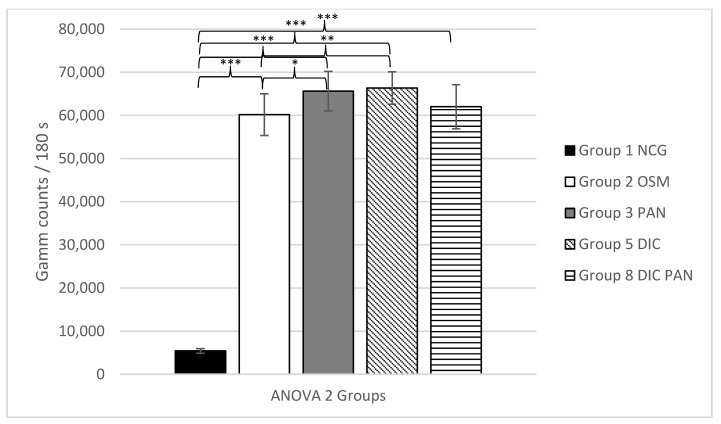
ANOVA 2, analyzing the ^99m^TC-HDP uptake (counts)/180 s for groups 1–3, 5 and 8 +/− standard deviation. Statistical significance is indicated by *** = *p* ≤ 0.001; ** = *p* ≤ 0.01, * = *p* ≤ 0.05.

**Figure 3:**
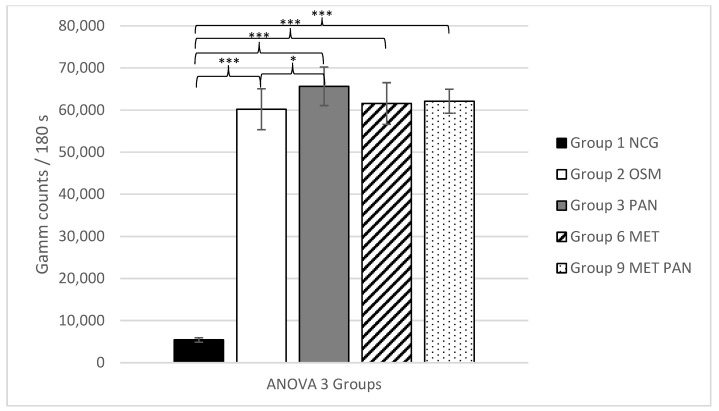
ANOVA 3, analyzing the ^99m^TC-HDP uptake (counts)/180 s for groups 1–3, 6 and 9 +/− standard deviation. Statistical significance is indicated by *** = *p* ≤ 0.001; * = *p* ≤ 0.05.

**Figure 4:**
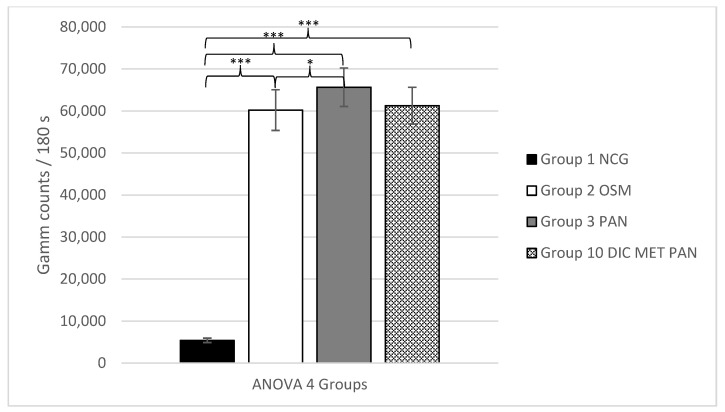
ANOVA 4, analyzing the ^99m^TC-HDP uptake (counts)/180 s for groups 1–3 and 10 +/− standard deviation. Statistical significance is indicated by *** = *p* ≤ 0.001; * = *p* ≤ 0.05.

The mean values of each group with the corresponding ANOVA analyses 1–4 are shown in Figure 1, Figure 2, Figure 3 and Figure 4.

## 3. Discussion

Musculoskeletal disorders require sufficient pain medication for a symptomatic treatment [2]. If a surgical treatment is necessary, whether this is an elective orthopaedic procedure (e.g., total hip replacement due to progredient arthrosis) or a trauma surgical emergency procedure (e.g., reduction and osteosynthesis due to a fracture), it is critical that, in the post-surgical setting, the patient is almost pain free to allow fast recovery, reduce morbidity and mortality, to avoid discomfort and to ensure immediate mobilization [4,7]. However, recent evidence indicates that certain (pain) medication may have an unknown effect on bone metabolism [20].

The most important finding of our study is that the NSAIDs tested, as well as the non-NSAID metamizole, do not have a negative effect on the hydroxyapatite synthesis of human MSCs, the key marker of osteogenesis, under in vitro conditions and so do not affect the osteogenic differentiation. While this study focused on the hydroxyapatite production by the hMSCs within the different groups, no direct evaluation of the potential impact of the drugs on the viability of the cells was performed. If any of the drugs or their combination caused a critical impact on the cell viability, a significantly lower production of hydroxyapatite would have been expected. However, the analysis of the mean uptake values of all groups (3–8) where one or more drugs were added showed a higher uptake of the tracer compared to group (2) where only the osteogenic supplements were added. Therefore, it can be assumed that none of the drugs affected the cell viability substantially. In all the ANOVA analyses performed, there was a highly significantly major uptake of the radioactive tracer (*p* ≤ 0.001) within all groups treated with OSM enriched with ibuprofen (group 4), diclofenac (group 5) and metamizole (group 6), as compared to the negative control group (group 1). These results concur with the results from previous in vitro studies that also revealed no negative effect on the osteogenic differentiation [22,23]. Normal levels of ibuprofen seem to have no impact on the osteogenic response of MSCs in vitro [22], while other data indicates that the osteogenic potential of MSCs is inhibited/delayed by the treatment of high-dose NSAIDs under inflammatory conditions [24]. Another study assumes a potential reduction in bone metabolism, as their data indicate an inhibition of the chondrogenesis that might affect enchondral bone formation by NSAIDs [23]. Based on this theory, there are some recommendations that high-risk patients should not be treated with NSAIDs [20]. As our in vitro model does not support enchondral bone formation, no statement can be made from our results regarding this thesis. Other in vitro studies indicate that the osteogenic marker alkaline phosphatase, bone sialoprotein, and osteocalcin, and chondrogenesis marker gene aggrecan can be negatively affected by certain pain drugs while diclofenac is the only NSAID that has no negative effect upon all of these osteogenic markers [25]. Our analysis showed not only that diclofenac has no negative effect upon osteogenesis, but also a significant positive impact on the in vitro osteogenesis, resulting in a clearly higher uptake (*p* ≤ 0.05) of the tracer in the group additionally treated with diclofenac (group 5) compared to the standard osteogenic group (group 2), even in our multi donor in vitro setting. This result is surprising from a clinical point of view, as diclofenac, as well as other NSAIDs, is known to suppress osteogenesis and reduce heterotopic ossifications [9,10]. Other in vitro data support our results, as it has been shown that diclofenac is the only NSAID that does not negatively affect the expression of the osteogenic key markers in MSCs [25]. Thus, there is evidence, supported by our results, that diclofenac supports in vitro osteogenic differentiation but the mechanism remains unknown. It is known, that BM-MSCs have compensatory mechanism to restore PEG2 synthesis as an intrinsic regulation of osteogenic differentiation if these cells are treated with NSAIDs [26] while the inhibition of the prostaglandin synthesis in vivo result is a limitation of pathologic bone growth, which makes these drugs very popular to avoid heterotopic ossifications after total hip replacement [10,11]. However, recent systematic reviews confirmed the absence of robust in vitro or clinical data regarding NSAIDs and their impact on bone formation, and encouraged researchers to perform further basic science and clinical studies to clarify the risks and benefits of NSAIDs in patients with musculoskeletal disorders [19]. Unfortunately, the direct hydroxyapatite content has never been evaluated, neither in the studies investigating in the effects of NSAID nor in the studies investigating in the effects of PPI upon the osteogenic differentiation in vitro. We define the absolute hydroxyapatite content as one of the most important parameters that should always be quantified directly in all in vitro studies regarding the effects on bone metabolism in vitro as it is a functional and direct assessment of the mineral. Previous in vitro data regarding the effect of PPIs on osteogenesis showed a negative impact, resulting in a deleterious effect on bone cells [16,27,28], while our data distinctly indicates a consistently beneficial effect on the osteogenesis by adding just PPIs to the cell cultures of MSCs (*p* ≤ 0.05). This might be a result of increased mitochondrial activity, as previously shown [17]. However, no significant positive effect on the osteogenesis could be shown by adding PPIs as a concomitant medication to the drugs ibuprofen, diclofenac or metamizole. It has to be noted that, within our experimental set-up, we investigated the PPI impact on in vitro osteogenesis by using only one PPI (Pantoprazole). Beside Pantoprazole, there various other PPIs available (Omeprazole, Esomeprazole, Dexlansoprazole, etc.) that all act by irreversibly blocking the hydrogen/potassium adenosine triphosphatase enzyme. Regarding their configuration PPIs can be generally divided into the benzimidazole group or the imidazopyridine, while all commercially available PPIs belong to the benzimidazole group. Nevertheless, as all of these drugs have a slightly different chemic structure, it cannot be assumed that the presented results can be generalized for all PPIs. Therefore, additional studies will be necessary to compare the different PPIs and their effect upon osteogenic differentiation in vitro.

Additionally, combining diclofenac and pantoprazole has no further positive effect on osteogenesis (*p* = 1), even though both drugs individually have a significant positive impact (*p* ≤ 0.05, *p* ≤ 0.01). The combination of diclofenac, metamizole and pantoprazole has no beneficial effect on the osteogenesis of hMSCs as well.

In summary, our in vitro data suggest no negative effect of the NSAIDs ibuprofen and diclofenac or the non-NSAID metamizole on the osteogenesis of MSCs, while the NSAID diclofenac and the PPI pantoprazole enhance the production of hydroxyapatite. No negative effect of any single drug or combination of drugs on bone metabolism in vitro could be revealed. Further, no additional positive effect was found by combining the pain-reliving drugs with the PPI pantoprazole.

As no basic research or clinical data is available concerning the effects of different NSAIDs and other pain drugs in combination with PPIs towards bone metabolism, especially not in the context of evaluating the hydroxyapatite content, there is a strong need to perform further studies in this field, as it is very important to treat the aging population with appropriate pain medication without the risk of negatively influencing their bone metabolism.

Limitation of the study: We use a standard monolayer cell culture model, so it has to be kept in mind that the physiological pathways in humans are far more complex and cannot be easily simulated. This is particularly the case for individual resorption kinetics of the drugs, leading to different serum blood levels in vivo. Additionally, only one agent of every drug type (e.g., pantoprazole as PPI) was used while there are a variety of other drugs with the same therapeutic purpose, but with slightly different chemical structures, are available on the market. Therefore, the results must be carefully interpreted and cannot be generalized to the whole drug family when they are transferred from the bench to the bedside. Subsequent studies will be performed using a three-dimensional cell culture model to emulate the bone formation in vivo while using different concentrations of the drugs.

## 4. Materials and Methods

### 4.1. Experimental Design at a Glance

Human mesenchymal stem cells (hMSC) were harvested from the femoral bone cavity of healthy donors (n = 4) followed by cell expansion in T-175 flasks. All donors were Caucasians, three male (age 67, 79, 72) and one female (age 67). The cells were seeded in 35 mm flat bottom petri dishes: donor 1 6-fold, donor 2 2-fold, donor 3 2-fold, donor 4 2-fold each in ten different groups. This resulted in 12 dishes from 4 different donors for each group, and 120 dishes for the entire experiment.

Group 1 Non-osteogenic negative control group (NCG)
Group 2 Osteogenic differentiation media without and drugs (OSM)
Group 3 OSM with pantoprazole (PAN)
Group 4 OSM with ibuprofen (IBU)
Group 5 OSM with diclofenac (DIC)
Group 6 OSM with metamizole (MET)
Group 7 OSM with ibuprofen and pantoprazole (IBU + PAN)
Group 8 OSM with diclofenac and pantoprazole (DIC + PAN)
Group 9 OSM with metamizole and pantoprazole (MET + PAN)
Group 10 OSM with diclofenac, metamizole and pantoprazole (DIC + MET + PAN)

The exact media formula for each group is described in Section 4.6 Preparation of Cell Culture Media and Drug Concentrations.

Dishes were cultured for 21 days in the appropriate cell culture media at 37 °C and 5% CO_2_ with media change every two days. The cell cultures were subsequently terminated and washed and then incubated with 5 MBq of the radioactive tracer ^99m^Tc-HDP. Gamma camera imaging/analysis and post-imaging software processing were then performed. During this analysis and processing, regions of interest were defined to calculate the exact amount of tracer bound to each dish. This reflected the amount of hydroxyapatite and hence the osteogenic potential of the cell culture media.

### 4.2. hMSC Harvest and Expansion

Bone marrow aspirates were obtained from the proximal femoral cavity of four healthy donors (n = 4) under general anaesthesia during an elective surgical procedure for total hip arthroplasty after informed consent and positive approval by the local ethics committee. During preparation of the proximal femoral bone cavity, 10–15 mL of bone marrow was collected into a 20 mL syringe (BD^®^) (San Jose, CA 95131, USA) containing 1000 IU heparin (5000 IU/mL; B-Braun^®^) (Melsungen, Germany). Individual samples were diluted 1:1 with phosphate-buffered saline (PBS; Invitrogen^®^) (Thermo Fisher Scientific, Waltham, MA, USA) and washed twice with PBS. The mononuclear cell fraction was isolated by Ficoll gradient centrifugation (Ficoll-Paque-PLUS; GE Healthcare^®^) (VWR International GmbH, Darmstadt, Germany). Mononuclear cells were plated in T-175 polystyrene tissue culture flasks (BD Falcon^®^) (Thermo Fisher Scientific, Waltham, MA, USA) at a density of 5 × 10^5^/cm^2^ and cultured in a humidified 5% CO2 atmosphere at 37 °C in low-glucose Dulbecco’s modified Eagle’s medium (DMEM-LG, Invitrogen^®^) (Thermo Fisher Scientific, Waltham, MA, USA) containing 10% heat inactivated (56 °C, 30 min) foetal bovine serum (FBS, Invitrogen^®^) (Thermo Fisher Scientific, Waltham, MA, USA) and 1% Penicillin/Streptomycin (Invitrogen^®^) (Thermo Fisher Scientific, Waltham, MA, USA). After 48 h, nonadherent cells were removed and the adherent cells were washed with PBS. Media was changed every 2–3 days. At 90% confluence, cells were trypsinized. For further experiments, these P0 cells were frozen in liquid nitrogen in 0.5 mL aliquots containing 5 × 10^5^ cells in DMEM-LG with 20% FBS and 10% DMSO (Sigma^®^St. Louis MO, USA).

### 4.3. Osteogenic Differentiation Assay

P0 human donor (n = 4) MSCs, frozen in N2 in 0.5ml aliquot vials, containing 5 × 10^5^ cells each, were thawed and seeded into T-175 flask (BD Falcon^®^) (Thermo Fisher Scientific, Waltham, MA, USA) with 250,000 cells each, and cultured for 10 days to harvest enough cells for the further experiments. Donor 1: 3 vials = 6 flasks, donor 2: 1 vial = 2 flasks, donor 3: 1 vial = 2 flasks, and donor 4: 1 vial = 2 flasks

During this cell expansion stage, Dulbecco’s modified Eagle’s medium (DMEM-LG, Invitrogen^®^) (Thermo Fisher Scientific, Waltham, MA, USA) containing 10% heat inactivated (56 °C, 30 min) foetal bovine serum (FBS, Invitrogen^®^Thermo Fisher Scientific, Waltham, MA, USA) and 1% Penicillin/Streptomycin (Invitrogen^®^(Thermo Fisher Scientific, Waltham, MA, USA) was used with media change every 2–3 days cultured in a humidified 5% CO2 atmosphere at 37 °C. After 10 days, approximately 80%–90% of confluence was reached and cells were trypsinized and resuspended, and cells from every donor were seeded at a density of 15,000 cells/cm^2^ into 35 mm flat bottom petri dishes (Corning^®^) (Corning NY, USA).

For every group (total of 10 groups) six dishes were seeded with cells from donor 1, two dishes were seeded with cells from donor 2, two dishes were seeded with cells from donor 3 and two dishes were seeded with cells from donor 4, resulting in a total of 120 dishes.

Immediately after seeding, the designated media was added to the dishes from each group.

Group 1 (NCG): Non-osteogenic, negative control group (NCG)

Group 2 (OSM): Standard osteogenic media (OSM)

Group 3 (PAN): OSM with pantoprazole

Group 4 (IBU): OSM with ibuprofen

Group 5 (DIC): OSM with diclofenac

Group 6 (MET): OSM with metamizole

Group 7 (IBU + PAN): OSM with ibuprofen and pantoprazole containing

Group 8 (DIC + PAN): OSM with diclofenac and pantoprazole

Group 9 (MET + PAN): OSM with metamizole and pantoprazole

Group 10 (DIC + MET + PAN): OSM with diclofenac, metamizole and pantoprazole

Cells were treated for 21 days with specific media change every 2–3 days, cultured in a humidified 5% CO2 atmosphere at 37 °C. The exact media formula for each group is described in Section 4.6.

### 4.4. ^99m^Tc-HDP Labelling

After termination of the cell cultures, the medium was removed and the dishes carefully washed twice with phosphate-buffered saline (PBS, Invitrogen^®^Thermo Fisher Scientific, Waltham, MA, USA). An aliquot of 5 MBq ^99m^Tc-HDP (Technescan-HDP^®^, Mallinckrodt Pharmaceuticals, Staines-upon Thames, UK)) in 2 mL of 0.9% NaCl solution was added to each dish. Technetium activity was assayed with a dose calibrator (Activimeter ISOMED 1010, Nuklear-Medizintechnik Dresden GmbH, Dresden, Germany). Dishes were then incubated at room temperature for 2 h. The remaining liquid ^99m^Tc-HDP was removed and the dishes were washed three times (for approximately 30 min) with 2 mL PBS each to remove the unbound tracer. Six blank dishes were incubated with the tracer in parallel to determine the mean amount of background uptake. The dishes were then placed directly on the detector of a gamma camera (E.CAM+, Siemens, Erlangen, Germany) with a 256 × 256 detector grid and the radionuclide counts were acquired for 180 s [21,29].

To determine the exact bound activity emitted by each dish, the acquisition software Xeleris^®^ 3 (GE Healthcare^®^ Chalfont St Giles, Buckinghamshire, UK) was used to define regions of interest (ROIs) around each dish within the detection area. Each ROI had exactly the same pixel size. The number of gamma counts for each ROI was calculated by the software, reflecting the amount of bound tracer and therefore the amount of hydroxyapatite. After imaging, the dishes were stored at room temperature for 72 h to allow ^99m^Tc-HDP to decay to background levels.

### 4.5. Statistics

SPSS Statistics^®^ Version 20, (IBM, Armonk, NY, USA) was used for the statistical analysis. Statistical significance was set to *p* ≤ 0.05. A Kolmogorov-Smirnov-Test for normal distribution was performed prior to further analysis of the data.

To reveal differences caused by the influence of the different media towards the osteogenic differentiation of the cells three one-factor ANOVA analyses with Bonferroni post-hoc testing were performed. Each ANOVA analyzed differences between the negative control group (group 1), the standard osteogenic group (group 2), the pantoprazole group (group 3), one of the standard pain drugs (group 4–6) and the standard pain drug + pantoprazole (group 7–9).

ANOVA 1: Group 1 NCG, group 2 OSM, group 3 PAN, group 4 IBU, group 7 IBU + PAN.

ANOVA 2: Group 1 NCG, Group 2 OSM, group 3 PAN, group 5 DIC, group 8 DIC + PAN.

ANOVA 3: Group 1 NCG, Group 2 OSM, group 3 PAN, group 6 MET, group 9 MET + PAN.

The fourth ANOVA was performed to analyze statistical differences between the negative control group (group 1), the standard osteogenic group (group 2), the pantoprazole group (group 3) and the group in which diclofenac was combined with metamizole and pantoprazole (group 10)

ANOVA 4: Group 1 NCG, Group 2 OSM, group 3 PAN, group 10 DIC + MET + PAN.

### 4.6. Preparation of Cell Culture Media and Drug Concentrations

All used drugs are certified by the German authority board (Federal Institute for drugs and medical devise, BfArM) for the human application. Reference for the applied drug concentrations in the cell culture media was taken from the pharmacokinetic and pharmacodynamic data supplied by the manufacturer corresponding to the concentrations in the human body.

Group 1 (NCG): Non-osteogenic, negative control group (NCG), containing DMEM-LG (Invitrogen^®^Thermo Fisher Scientific, Waltham, MA, USA), 10% foetal bovine serum (FBS, Invitrogen^®^Thermo Fisher Scientific, Waltham, MA, USA) and 1% Penicillin/Streptomycin (Invitrogen^®^ Thermo Fisher Scientific, Waltham, MA, USA).

Group 2 (OSM): OSM containing DMEM-LG (Invitrogen^®^ Thermo Fisher Scientific, Waltham, MA, USA), 10% foetal bovine serum (FBS, Invitrogen^®^ Thermo Fisher Scientific, Waltham, MA, USA) and 1% Penicillin/Streptomycin (Invitrogen^®^ Thermo Fisher Scientific, Waltham, MA, USA) and the osteogenic supplements 100 nM dexamethasone (Sigma^®^ St. Louis MO, USA), 10 mM ß-glycerol phosphate (Sigma^®^ St. Louis MO, USA), and 0.173 mM L-ascorbic acid-2-phosphate (Wako^®^ FUJIFILM Wako Chemicals Europe GmbH, Neuss, Germany) [30].

Group 3 (PAN): OSM with pantoprazole, containing (OSM) and 1.5 µg/mL pantoprazole (Pantozol^®^, TAKEDA GmbH, Berlin, Germany) [31].

Group 4 (IBU): OSM with ibuprofen, containing (OSM) and 30 µg/mL ibuprofen (Ibuprofen ratiopharm^®^, ratiopharm GmbH, Ulm, Germany) [22].

Group 5 (DIC): OSM with diclofenac containing (OSM) and 1 µg/mL diclofenac (Voltaren dispers^®^, Novartis AG, Basel, Swiss) [23].

Group 6 (MET): OSM with metamizole, containing (OSM) and 17.3 µg/mL metamizole (Novalgin^®^, Sanofi-Aventis GmbH, Frankfurt am Main, Germany) [32].

Group 7 (IBU + PAN): OSM with ibuprofen and pantoprazole containing (OSM) and 30 µg/mL ibuprofen (Ibuprofen ratiopharm^®^, ratiopharm GmbH) [22] and 1.5 µg/mL pantoprazole (Pantozol^®^, TAKEDA GmbH, Berlin, Germany) [31].

Group 8 (DIC + PAN): OSM with diclofenac and pantoprazole containing (OSM), 1 µg/mL diclofenac (Voltaren dispers^®^, Novartis AG, Basel, Swiss) [23] and 1.5 µg/mL pantoprazole (Pantozol^®^, TAKEDA GmbH, Berlin, Germany) [31].

Group 9 (MET + PAN): OSM with metamizole and pantoprazole containing (OSM), 17.3 µg/mL metamizole (Novalgin^®^, Sanofi-Aventis GmbH, Basel, Swiss) [32] and 1.5 µg/mL pantoprazole (Pantozol^®^, TAKEDA GmbH, Berlin, Germany) [31].

Group 10 (DIC + MET + PAN): OSM with diclofenac, metamizole and pantoprazole containing (OSM), 1 µg/mL diclofenac (Voltaren dispers^®^, Novartis AG) [23], 17.3 µg/mL metamizole (Novalgin^®^, Sanofi-Aventis GmbH, Frankfurt am Main, Germany) [32] and 1.5 µg/mL pantoprazole (Pantozol^®^, TAKEDA GmbH) [31].

## Figures and Tables

**Table 1:** Mean, minimum and maximum gamma counts/180 s for each group.

Group	N	Mean	Minimum	Maximum	Standard Deviation
Group_1_NCG	12	328	2	1170	322
Group_2_OSM	12	54,880	46,194	60,107	5061
Group_3_PAN	12	60,333	46,627	64,402	4791
Group_4_IBU	12	59,124	49,896	63,246	4403
Group_5_DIC	12	61,048	51,602	65,606	3939
Group_6_MET	12	56,246	45,070	60,775	5159
Group_7_IBU_PAN	12	58,624	50,548	62,402	3859
Group_8_DIC_PAN	12	56,694	43,372	64,111	5349
Group_9_MET_PAN	12	56,778	50,133	60,326	2972
Group_10_DIC_MET_PAN	12	55,940	47,865	61,387	4562

## Data Availability

Full dataset of all study data can be obtained upon request.

## Abbreviations

ANOVA	Analysis of variance
Bq	Becquerel
BM	Bone marrow
COX-1	Cyclooxygenase-1
COX-2	Cyclooxygenase-2
DIC	diclofenac
hMSC	Human mesenchymal stem cells
IBU	ibuprofen
MET	metamizole
MeV	Mega-electron volts
MSC	Mesenchymal stem cells
NSAID	nonsteroidal anti-inflammatory drugs
OSM	Osteogenic treatment group
NCG	Non-osteogenic, negative control group
OSM	Standard osteogenic media
PAN	pantoprazole
PEG2	Prostaglandine 2
PPI	Protone pump inhibitor
ROI	Region of interest
WHO	World Health Organistaion
